# Safety and efficacy of balloon pulmonary angioplasty in chronic thromboembolic pulmonary hypertension in the Netherlands

**DOI:** 10.1007/s12471-019-01352-6

**Published:** 2019-11-28

**Authors:** M. C. J. van Thor, R. J. Lely, N. J. Braams, L. ten Klooster, M. A. M. Beijk, R. H. Heijmen, D. A. F. van den Heuvel, B. J. W. M. Rensing, R. J. Snijder, A. Vonk Noordegraaf, E. J. Nossent, L. J. Meijboom, P. Symersky, J. J. Mager, H. J. Bogaard, M. C. Post

**Affiliations:** 1 Department of Cardiology, St. Antonius Hospital, Nieuwegein, The Netherlands; 2 Department of Pulmonary Medicine, St. Antonius Hospital, Nieuwegein, The Netherlands; 3 Department of Cardiothoracic surgery, St. Antonius Hospital, Nieuwegein, The Netherlands; 4 Department of Radiology, St. Antonius Hospital, Nieuwegein, The Netherlands; 5 Department of Cardiology, Amsterdam UMC, Vrije Universiteit Amsterdam, Amsterdam, The Netherlands; 6 Department of Pulmonary Medicine, Amsterdam UMC, Vrije Universiteit Amsterdam, Amsterdam, The Netherlands; 7 Department of Cardiothoracic surgery, Amsterdam UMC, Vrije Universiteit Amsterdam, Amsterdam, The Netherlands; 8 Department of Radiology and Nuclear Medicine, Amsterdam UMC, Vrije Universiteit Amsterdam, Amsterdam, The Netherlands

**Keywords:** Chronic thromboembolic pulmonary hypertension, Balloon pulmonary angioplasty, Safety, Clinical outcome, Efficacy

## Abstract

**Background:**

Balloon pulmonary angioplasty (BPA) is an emerging treatment in patients with chronic thromboembolic pulmonary hypertension (CTEPH) and chronic thromboembolic disease (CTED). We describe the first safety and efficacy results of BPA in the Netherlands.

**Methods:**

We selected all consecutive patients with inoperable CTEPH and CTED accepted for BPA treatment who had a six-month follow-up in the St. Antonius Hospital in Nieuwegein and the Amsterdam University Medical Center (UMC) in Amsterdam. Functional class (FC), N‑terminal pro-brain natriuretic peptide (NT-proBNP), 6‑minute walking test distance (6MWD) and right-sided heart catheterisation were performed at baseline and six months after last BPA. Complications for each BPA procedure were noted.

**Results:**

A hundred and seventy-two BPA procedures were performed in 38 patients (61% female, mean age 65 ± 15 years). Significant improvements six months after BPA treatment were observed for functional class (63% FC I/II to 90% FC I/II, *p* = 0.014), mean pulmonary artery pressure (−8.9 mm Hg, *p* = 0.0001), pulmonary vascular resistance (−2.8 Woods Units (WU), *p* = 0.0001), right atrial pressure (−2.0 mm Hg, *p* = 0.006), stroke volume index (+5.7 ml/m^2^, *p* = 0.009) and 6MWD (+48m, *p* = 0.007). Non-severe complications occurred in 20 (12%) procedures.

**Conclusions:**

BPA performed in a CTEPH expert centre is an effective and safe treatment in patients with inoperable CTEPH.

## What’s new

- Balloon pulmonary angioplasty in inoperable chronic thromboembolic pulmonary hypertension significantly improves symptoms, exercise capacity and pulmonary haemodynamics.
- Balloon pulmonary angioplasty is safe; no major complications and a 12% nonsevere complication rate.

## Introduction

Chronic thromboembolic pulmonary hypertension (CTEPH) is a pulmonary artery disease caused by chronic thromboembolisms, leading to pulmonary hypertension despite the use of anticoagulation. The pulmonary vascular resistance (PVR) increases due to vascular occlusions and a consequential distal arteriopathy, eventually resulting in right ventricular failure and death if left untreated [1].

When CTEPH patients are technically operable, i.e. in central or segmental disease, pulmonary endarterectomy is the treatment of choice [2, 3]. Patients who are considered inoperable, based on a distal localisation of chronic thromboembolisms or due to comorbidities resulting in a nonacceptable risk-benefit ratio, are currently treated with pulmonary hypertension specific medication [3, 4]. However, recent studies reported that balloon pulmonary angioplasty (BPA) might be of value in carefully selected patients [5]. Treatment with BPA aims to restore blood flow in the pulmonary arteries by opening partially or fully blocked vessels leading to improved pulmonary haemodynamics [5]. In addition, patients with symptomatic chronic thromboembolic disease (CTED) without pulmonary hypertension, may also benefit from BPA [6].

We evaluated the safety and efficacy of BPA treatment in inoperable CTEPH and CTED patients and present the first results of the two specialised CTEPH centres in the Netherlands.

## Methods

### Study population

Multidisciplinary CTEPH teams diagnosed CTEPH and assessed operability in accordance with the current guideline [4]. The teams consist of (interventional) cardiologists, pulmonologists, (interventional) radiologists, cardiothoracic surgeons and specialised nurse practitioners. Pulmonary hypertension was defined as a mean pulmonary artery pressure (mPAP) of ≥25 mm Hg and a pulmonary artery wedge pressure (PAWP) ≤15 mm Hg on right-sided heart catheterisation. The diagnosis of CTEPH was established with mismatched perfusion defects on lung scan in the presence of specific diagnostic signs for CTEPH on multidetector computed tomography angiography or conventional pulmonary angiography, after anticoagulation for a minimum of three months. Symptomatic patients with perfusion defects, but with a mPAP <25 mm Hg, were classified as CTED.

Pulmonary hypertension specific medical therapy was initiated in case of symptomatic, inoperable CTEPH. Patients remained on the same therapy during BPA procedures, unless a change in therapy was clinically necessary.

All patients were discussed in the CTEPH team. Inoperable CTEPH/CTED patients or patients reluctant to undergo surgery were accepted for BPA treatment if they had accessible thromboembolic lesions and did not have severe contraindications (i.e. right-sided valvular endocarditis, right-sided mechanical heart valve or the presence of thrombus or myxoma in the right atrium). Patients with relative contraindications (i.e. patients with an estimated creatinine clearance <30 ml/min/1.73m^2^, hypersensitivity to contrast media, coagulopathy or pregnancy) were individually reviewed. Patients accepted for BPA treatment, were enrolled in a standardised BPA (follow-up) protocol, including BPA treatment and a follow-up evaluation six months after the last BPA. All patients accepted for BPA in the St. Antonius Hospital in Nieuwegein between January 2016 and October 2018, and in the Amsterdam UMC between June 2015 and February 2019, were included in this study. Data from all patients who completed their six-month follow-up before the end of the study period were used for statistical analyses.

### BPA procedure and right-sided heart catheterisation St. Antonius Hospital

**Fig. 1:**
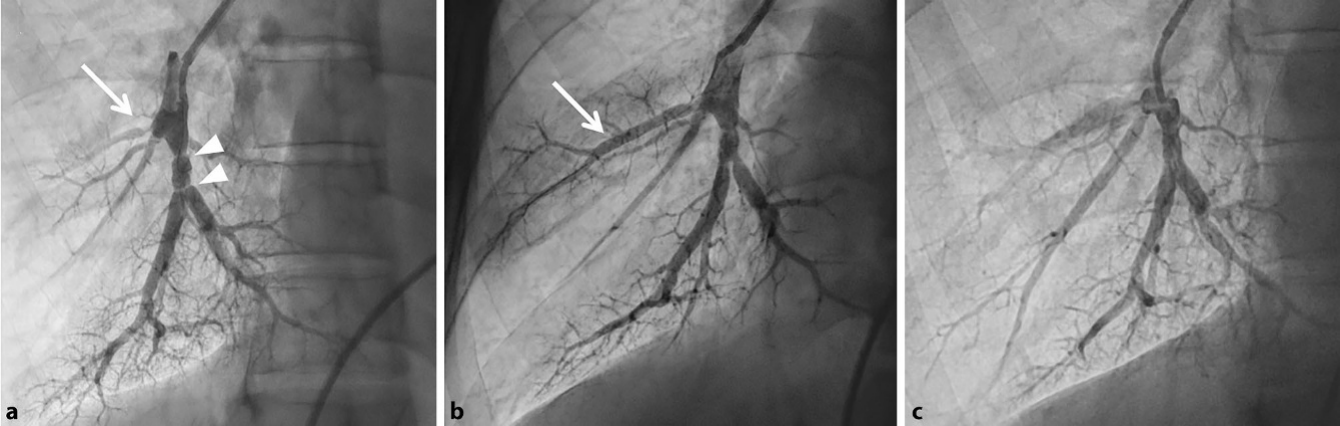
Three images of the right lower and middle pulmonary artery lobe on pulmonary angiography in the same CTEPH patient. **a** Pre BPA, with significant lesions in the middle lobe (total occlusion, *white arrow*) and lower lobe (webs, *white arrow heads*). **b** Result after the first BPA procedure, with opening of the total occlusion in the middle lobe. **c** Result after the second BPA procedure, with dilatation of web lesions in the lower lobe. *CTEPH* chronic thromboembolic pulmonary hypertension, *BPA* balloon pulmonary angioplasty

Oral anticoagulation was maintained throughout all interventions with an international normalised ratio (INR) between 2.5 and 3.5. Patients on direct oral anticoagulants (DOACs) were actively switched to vitamin K antagonists to minimise the risk of procedure-related thromboembolisms. Right-sided heart catheterisation was performed with a Swan-Ganz 7F catheter, at baseline and six months after the last BPA procedure. BPA procedures were performed using femoral access with a 6F to 9F sheath and a 6F guide wire and catheter (Terumo Corporation, Tokyo, Japan). Activated clotting time was kept between 200 and 300 seconds by intravenous heparin therapy (2500–5000 IU). The 0.014-inch guide wire was directed into the affected pulmonary artery branches, which were visualised with iopromide (ULTRAVIST). After identification of the affected vessels and correct positioning of the guide wire, dilatation with semi-compliant balloons (Emerge 2.5–4.0/15–20 mm or Maverick XL 5.0/15–20 mm, Boston Scientific, Marlborough, MA and Tazuna RX 3.0/20 mm, Terumo Corporation, Tokyo, Japan) was performed. During each procedure, up to four vessels were treated or the procedure ended when more than 500 ml contrast or more than 60 minutes procedure time was used. Time to the next BPA procedure varied between four to six weeks. Vital signs and signs of potential complications were monitored at the coronary care unit after each procedure. Fig. 1 shows images and results of BPA in an inoperable CTEPH patient.

### BPA procedure and right-sided heart catheterisation Amsterdam UMC

Vitamin K antagonists were stopped at least two weeks prior to the procedure and were switched to DOACs. Patients stopped antiplatelet medication at least one week before the procedure. Depending on the renal clearance, anticoagulation was stopped 24–48 hours prior to BPA. The procedure took place if the INR was ≤1.5 on the day of procedure. Right-sided heart catheterisation was performed with a Swan-Ganz 8F catheter during the diagnostic pulmonary angiogram, and six months after the last BPA procedure. BPA procedures were performed using femoral access with a 6F Destination sheath (Terumo) and a 6F guiding catheter (Cordis Corporation, Miami Lakes, FL USA and Medtronic Inc, Medtronic Parkway, Minneapolis, MN USA). Activated clotting time was kept between 250 and 350 seconds by intravenous heparin therapy (starting with 5000 IU). A 0.014-inch guide wire was directed into the affected pulmonary artery branches, which were visualised with iodixanol (Visipaque 320). Dilatation was performed with semi-compliant balloons (Emerge 2.0–3.0/20 mm, Maverick XL 5.0/15 mm, Boston Scientific, Marlborough, MA USA and TREK 3.5–4.0/20 mm, Abbott Vascular, Santa Clara, CA USA). The procedure ended if a maximum of ten segments in one lung were treated or more than 200 ml contrast was used. Time to the next BPA procedure varied between four to six weeks. Patients were monitored for potential complications at the coronary care unit or pulmonary ward.

### Baseline and follow-up

We defined baseline as the date of CTEPH diagnosis in our multidisciplinary CTEPH teams. Additional research with imaging tests, right-sided heart catheterisation, World Health Organisation functional class (WHO FC), N‑terminal pro-brain natriuretic peptide (NT-proBNP) and 6‑minute walking test distance (6MWD) was performed at baseline and six months after the last BPA. Complications for each BPA procedure were noted.

### Statistical analyses

Statistical analyses were performed with SPSS software (IBM SPSS statistics version 24). Tests were 2‑tailed and with a *p*-value <0.05 considered statistically significant. Continuous data were presented as mean ± standard deviation (SD) or as median with interquartile range (IQR), and categorical data as number and percentage. Differences between baseline and six-month follow-up after BPA were assessed with the Wilcoxon signed-rank test, McNemar test and the exact McNemar test. The study was approved by the local medical ethical commissions in the St. Antonius Hospital (number W17.132) and Amsterdam UMC (number 2017.400).

## Results

### Study population

**Fig. 2:**
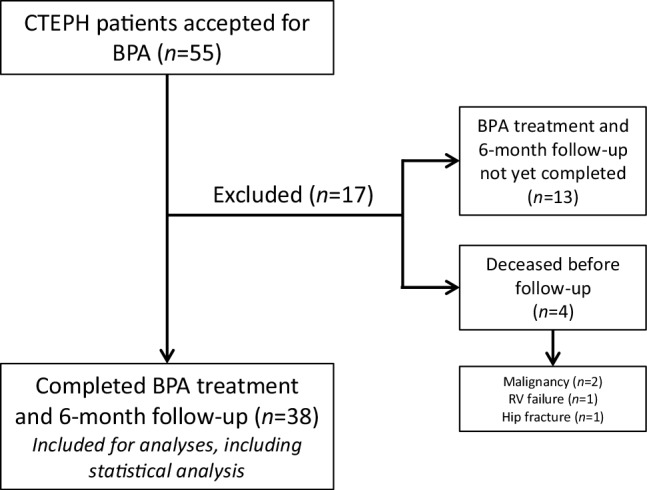
Flow chart with patient inclusion and exclusion. *CTEPH* chronic thromboembolic pulmonary hypertension, *BPA* balloon pulmonary angioplasty, *RV* right ventricular

We included 55 CTEPH/CTED patients accepted for BPA in this study, of which 38 had completed their treatment and six-month follow-up before the end of the study period. Seventeen patients were excluded from statistical analyses because these patients had not reached their six-month follow-up yet (*n* = 4), died before the six-month follow-up evaluation (*n* = 4) or were still undergoing BPA procedures at the end of the study period (*n* = 9). The four patients in the Amsterdam UMC who died before their six-month follow-up, died due to metastatic cancer (*n* = 2; diagnosed after final BPA), hip fracture (*n* = 1) and right ventricular failure (*n* = 1) (Fig. 2).

Thirty-eight patients (St. Antonius Hospital *n* = 15, Amsterdam UMC *n* = 23) were included for analyses in this study. The mean age was 65.0 ± 14.6 years, 61% was female and 63% of patients were in WHO FC II (Tab. 1). Twenty-six (69%) patients had technically inoperable disease (inaccessible for pulmonary endarterectomy), 7 (18%) patients had operable disease with comorbidities resulting in a nonacceptable risk-benefit ratio for pulmonary endarterectomy, and 5 (13%) patients were operable, but had declined pulmonary endarterectomy. None of the included patients had BPA after pulmonary endarterectomy. One female patient (40 years old, WHO FC III, mPAP 22 mm Hg and PVR 1.6 WU) suffered from severely symptomatic CTED. There were no patients with a history of chronic osteomyelitis, inflammatory bowel disease, ventriculoatrial shunt or cardiac device. Furthermore, most patients (87%) did have a history of a prior thromboembolic event and four (11%) patients had a coagulation disorder (polycythaemia vera *n* = 3, factor V Leiden *n* = 1). Two (5%) patients had concomitant coronary artery disease.

**Table 1:** Patient baseline characteristics pre and post BPA

	Pre BPA (*n* = 38)	Post BPA (*n* = 38)	*P*-value
*Demographic characteristics*			
Age (years)	65.0 ± 14.6		
Female gender, *n* (%)	23 (61)		
Length (cm)	173.6 ± 9.2		
Weight (kg)	83.6 ± 17.8		
BMI (kg/m^2^)	27.9 ± 6.2		
BSA (m^2^)	2.0 ± 0.2		
*Medical history, n (%)*			
Smokers (ever)	11 (29)		
COPD	6 (16)		
Hypertension	11 (29)		
Diabetes	3 (8)		
Coronary artery disease	2 (5)		
Thyroid dysfunction	3 (8)		
Coagulation disorder	4 (11)		
Venous thromboembolism	33 (87)		
*Pharmacologic therapy,n* (%)			
VKA/NOAC	15 (39)/23 (61)		
Diuretics	14 (37)		
No PH medication	7 (18)	5 (13)	0.500
sGC stimulator	8 (21)	10 (26)	0.500
PDE5‑i	3 (9)	4 (11)	1.000
ERA	5 (13)	8 (21)	0.250
sGC stimulator + ERA	7 (18)	6 (16)	1.000
PDE5-i + ERA	8 (21)	5 (13)	0.250
*Clinical characteristics*			
WHO FC I/II/III/IV (%)	0/63/34/3	39/50/11/0	*0.014*
NT-proBNP (pg/ml), median (IQR), *n* = 36	195 (96–1812)	154 (71–387)	0.078
Log NT-proBNP (pg/ml), *n* = 36	2.5 ± 0.8	2.2 ± 0.6	*0.008*
6MWD (m), *n* = 31	374 ± 124	422 ± 125	*0.007*
*Right-sided heart catheterisation*			
Heart rate (bpm), *n* = 31	72.3 ± 11.6	71.6 ± 11.8	0.630
CO (l/min), *n* = 36	5.7 ± 2.3	6.0 ± 1.6	0.510
CI (l/min/m^2^), *n* = 36	2.9 ± 1.1	3.0 ± 0.8	0.479
SVi (ml/m^2^), *n* = 31	37.5 ± 10.9	43.2 ± 10.2	*0.009*
Mean RAP (mm Hg), *n* = 33	8.9 ± 3.5	6.9 ± 3.0	*0.006*
Systolic PAP (mm Hg)	63.6 ± 19.8	50.6 ± 15.0	*0.0001*
Diastolic PAP (mm Hg)	24.1 ± 6.1	18.4 ± 4.2	*0.0001*
Mean PAP (mm Hg)	39.5 ± 11.6	30.6 ± 8.2	*0.0001*
PAWP (mm Hg)	11.5 ± 2.7	12.1 ± 3.6	0.340
PVR (WU), *n* = 36	6.1 ± 4.7	3.3 ± 2.0	*0.0001*

Data are presented as mean ± standard deviation or median (interquartile range)

*BPA* balloon pulmonary angioplasty, *SD* standard deviation, *BMI* body mass index, *BSA* body surface area, *COPD* chronic obstructive pulmonary disease, *VKA* vitamin K antagonist, *NOAC* novel oral anticoagulants, *PH* pulmonary hypertension, *sGC* soluble guanylyl cyclase, *PDE5‑i* phosphodiesterase type 5 inhibitor, *ERA* endothelin receptor antagonist, *WHO FC* World Health Organisation functional class, *NT-proBNP* N-terminal pro-brain natriuretic peptide, *IQR* interquartile range, *6MWD* 6-minute walking distance, *CO* cardiac output, *CI* cardiac index, *SVi* stroke volume index, *RAP* right arterial pressure, *PAP* pulmonary artery pressure, *PAWP* pulmonary artery wedge pressure, *PVR* pulmonary vascular resistance

## BPA procedure and complications

In total, 172 BPA sessions were performed (mean 4.5 ± 1.3 BPA sessions per patient). The total hospital stay was between 1.5 and 2.1 days per BPA session. Twenty (12%) complications were recorded (Tab. 2). Complications included mild haemoptysis (*n* = 14), temporary conduction or rhythm disturbances (*n* = 3), pulmonary vascular dissection (*n* = 2) and pulmonary vascular perforation (*n* = 1). There were no patients with vascular access complications in our cohort. Overall complication rates were similar between the two hospitals (St. Antonius Hospital 10% and Amsterdam UMC 12%). Mild haemoptysis was observed in 4% and 10% of all procedures in St. Antonius Hospital and Amsterdam UMC respectively (difference not statistically significant), while temporary conduction or rhythm disturbances were only observed in the St. Antonius Hospital.

**Table 2:** BPA-related complications

	Total BPA procedures (*n* = 172)
*Overall complications*	*20 (12)*
Mild haemoptysis	14 (8)
Temporary conduction/rhythm disturbances	3 (2)
Pulmonary vascular dissection	2 (1)
Pulmonary vascular perforation	1 (1)

*BPA* balloon pulmonary angioplasty

Multiple patients experienced haemoptysis during one BPA session (with the exception of one patient who experienced haemoptysis during three different BPA sessions), without angiographic vessel rupture, perforation or dissection. Haemoptysis not ending spontaneously was treated in two patients with intravascular pulmonary balloon occlusion of the vessel for 1–15 minutes and infusion of protamine. The two cases of pulmonary vascular dissection and the case with the pulmonary vascular perforation, all in subsegmental pulmonary arteries, were treated successfully in a similar way. In one patient, continuous positive airway pressure was deemed clinically necessary after BPA. None of the complications resulted in a longer hospital stay, death or need for intubation.

## Follow-up results

**Fig. 3:**
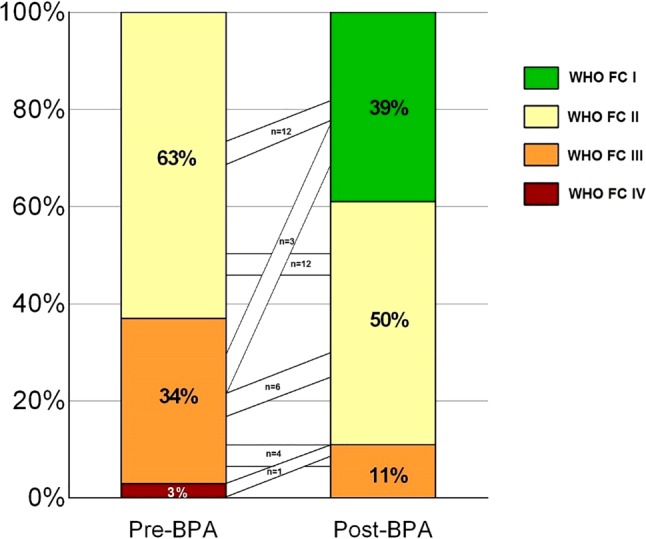
Change and results for WHO functional class pre BPA and six months post BPA. *WHO FC* World Health Organisation functional class, *BPA* balloon pulmonary angioplasty

The six-month follow-up after BPA was available in 38 patients and showed, compared with baseline, an improvement in WHO FC (63% FC I/II to 90% FC I/II, *p* = 0.014), with 58% of the patients improving at least one FC (Fig. 3), and increased 6MWD (+48 metres, *p* = 0.007). The median NT-proBNP did not significantly decrease, but the log NT-proBNP did (−0.3 log pg/ml, *p* = 0.008).

**Fig. 4:**
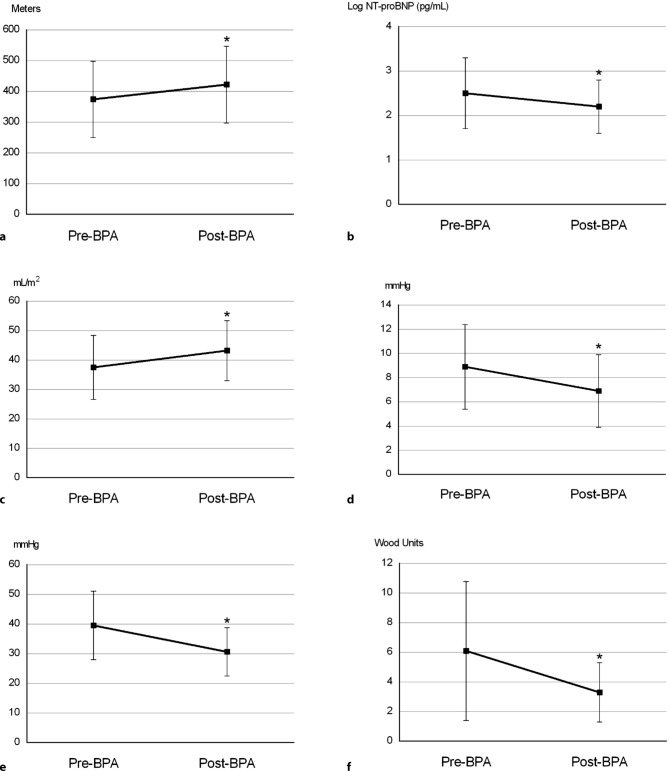
Results pre BPA and six months post BPA for **a** 6MWD, **b** log NT-proBNP, **c** SVi, **d** mRAP, **e** mPAP and **f** PVR. * indicates *p* < 0.05. *BPA* balloon pulmonary angioplasty, *6MWD* 6-minute walking distance, *NT-proBNP* N-terminal pro-brain natriuretic peptide, *SVi* stroke volume index,* mPAP* mean pulmonary artery pressure,* mRAP* mean right atrial pressure,* PVR* pulmonary vascular resistance

The systolic PAP (−13.0 mm Hg, *p* = 0.0001), diastolic PAP (−5.6 mm Hg, *p* = 0.0001) and mPAP (−8.9 mm Hg, *p* = 0.0001) significantly decreased, without a significant change in cardiac output and PAWP. This resulted in a significant improvement in PVR of −2.8 WU (*p* = 0.0001). Furthermore, stroke volume index (SVi) and right atrial pressure significantly improved (+5.7 ml/m^2^, *p* = 0.009 and −2.0 mm Hg, *p* = 0.006 respectively) (Fig. 4).

At baseline, 23 (61%) patients were using DOACs and 14 (37%) patients received concomitant loop diuretic therapy. At six-month follow-up, 87% of the patients were on pulmonary hypertension specific therapy and 7 patients (18%) had a change in their pulmonary hypertension specific therapy compared with baseline. Two patients started pulmonary hypertension specific therapy (riociguat *n* = 1 and bosentan *n* = 1), while five patients switched from combination therapy at baseline to monotherapy during follow-up due to clinical or haemodynamic improvement (Tab. 1).

The patient with CTED showed better values of 6MWD (+30m) and mPAP (−6.0 mm Hg), but without improvement in WHO FC (FC III).

## Discussion

Historically, the first BPA was performed in 1988 in Leiden, the Netherlands [7]. Results of the first series of patients who underwent BPA were reported in 2001, but due to a high percentage of severe complications, BPA was not used for years [8]. After several years, however, the technique was reintroduced in Japan. Since then, BPA has gained more interest and is now being used in many countries around the world [5]. The St. Antonius Hospital, Nieuwegein, Utrecht, and the Amsterdam UMC, Amsterdam, are the two CTEPH centres in the Netherlands where BPA procedures are performed. In this study we present the safety and efficacy results of BPA treatment in inoperable CTEPH patients. The main findings of the study are: 1) BPA is a safe and effective treatment in patients with inoperable CTEPH, 2) BPA significantly improves symptoms, exercise capacity and pulmonary haemodynamics. This is the largest cohort of patients evaluated in the Netherlands.

Complications occurred in 12% of all procedures, but without any severe consequences. Our complication rate was comparable with the complication rate reported in France—the largest single-centre BPA experience outside Japan—(initial period 16%), and Germany (9%) [9, 10]. However, it was slightly lower compared with results from large Japanese cohorts [5]. Our baseline pulmonary haemodynamics were slightly better compared with the other studies, as haemodynamics at baseline are predictors for outcome after BPA [5], this might partly explain the difference in complication rate.

Three episodes of temporary conduction and rhythm disturbances were observed after BPA, which resolved spontaneously; so clinical importance was low. It is possible that these abnormalities were coincidental findings already present before BPA. However, electrocardiograms at admission did not show these abnormalities. Conduction or rhythm disturbances have been described as complications of right-sided heart catheterisation procedures [11], but are rarely reported in other BPA studies. Nevertheless, they can become clinically important when they do not resolve spontaneously.

Haemoptysis, on the other hand, is often reported as a consequence of pulmonary artery injury and can be treated with balloon sealing by prolonged, low-pressure dilatation, slow infusion of protamine, embolisation or stenting [5]. Although we did not see severe pulmonary artery injury in the patients with haemoptysis during angiographic evaluation, except for one pulmonary vascular perforation, it is likely that the haemoptysis was caused by small pulmonary vascular injuries. It is striking that one patient experienced haemoptysis during three different BPA procedures, but this patient had severe impaired pulmonary haemodynamics (mPAP 50 mm Hg and PVR 12 WU), which may explain the increased risk and occurrence of complications. The usual treatments with balloon occlusion of the vessel, protamine infusion and continuous positive airway pressure were also effective in our patients.

The significant improvement in functional class (63% to 90% FC I/II) and exercise capacity (6MWD +48m) was comparable with results (35% to 79% FC I/II, 6MWD +45m) reported in the French study [9] and were slightly better than results (15% to 73% FC I/II, 6MWD +33m) reported in the German study [10], although in that study patients had more symptomatic and severe disease at baseline. Our results were comparable with the Japanese cohorts [5]. In accordance to other studies, the log NT-proBNP decreased significantly after BPA [12].

The baseline pulmonary haemodynamics in our patients were slightly better and the decrease in mPAP after BPA treatment (−9 mm Hg) was slightly lower compared with the French study (−12 mm Hg), although the change in PVR was similar (both −3 WU) [9]. This lower decrease in mPAP is probably related to the already better haemodynamic baseline values, as this may indicate less severe, and possibly treatable, CTEPH disease in the presence of a normal cardiac output. A Japanese multicentre registry showed a more pronounced decrease in post-procedural mPAP (43 to 22 mm Hg) [13]. However, the Japanese have more experience and perform BPA in different CTEPH patients [14].

In terms of clinical and haemodynamic improvement, results of BPA are lower than previous pulmonary endarterectomy results (6MWD +97m, mPAP −20 mm Hg and PVR −7 WU) [2], emphasising the importance of pulmonary endarterectomy as first choice in operable patients.

The SVi predicts outcomes in pulmonary arterial hypertension. Furthermore, SVi increased during treatment with pulmonary hypertension specific treatment in CTEPH (31 to 43 ml/m^2^) [15–17]. We report an increase in SVi (38 to 43 ml/m^2^) after BPA as an addition to pulmonary hypertension specific medical therapy, as well.

We performed BPA in one symptomatic CTED patient. Benefit from BPA treatment in CTED was shown previously, with improved functional class, exercise capacity and pulmonary haemodynamics [6]. Several parameters improved after BPA in our CTED patient, unfortunately without improvement in functional class. This might be explained by the fact that this patient had other severe comorbidities. More research about the effectiveness of BPA in CTED is necessary.

Healthcare costs are becoming increasingly important these days, with the total costs of five BPA procedures in the St. Antonius Hospital currently slightly cheaper than one year of riociguat treatment.

## Limitations

There was a different anticoagulation strategy in our two CTEPH centres. There are currently no guidelines/protocols about anticoagulation therapy during BPA, and so our hospitals made their own local protocols, based on their clinical practice. However, there needs to be a balance between bleeding complications and re-thrombosis in both strategies.

There were two (5%) patients in whom pulmonary hypertension specific therapy was initiated after baseline assessment. As pulmonary hypertension specific medical therapy also improves clinical outcomes (6MWD +3–51m) and haemodynamics (mPAP −5 mm Hg, PVR −2 WU) [18–22], our results may slightly overestimate the effect of BPA alone. However, there were five patients in whom dual pulmonary hypertension specific therapy could be downgraded to monotherapy. Trials about pulmonary hypertension specific medical therapy versus BPA in CTEPH are currently ongoing.

As we are specialised referral hospitals for BPA treatment in CTEPH, there may be a referral bias in patients referred for BPA (e.g. severely symptomatic or haemodynamically compromised patients not referred or patients incompletely evaluated for BPA and therefore not referred).

## Conclusion

BPA in inoperable CTEPH has a low complication rate and results in a significant improvement in symptoms, exercise capacity and pulmonary haemodynamics, when performed in a CTEPH expert centre.
